# DesA Prognostic Risk Model of LncRNAs in Patients With Acute Myeloid Leukaemia Based on TCGA Data

**DOI:** 10.3389/fbioe.2022.818905

**Published:** 2022-02-21

**Authors:** Weidong Ding, Yun Ling, Yuan Shi, Zhuojun Zheng

**Affiliations:** ^1^ Department of Hematology, The Third Affiliated Hospital of Soochow University, Soochow, China; ^2^ Laboratory of Hematology, The Third Affiliated Hospital of Soochow University, Soochow, China

**Keywords:** acute myeloid leukaemia, cox regression, lncRNA, prognostic risk model, TCGA

## Abstract

**Purpose:** This study aimed to combine the clinical data of acute myeloid leukaemia (AML) from The Cancer Genome Atlas (TCGA) database to obtain prognosis-related biomarkers, construct a prognostic risk model using long non-coding RNAs (lncRNAs) in AML and help patients with AML make clinical treatment decisions.

**Methods:** We analysed the transcriptional group information of 151 patients with AML obtained from TCGA and extracted the expressions of lncRNAs. According to the mutation frequency, the patients were divided into the high mutation group (genomic unstable group, top 25% of mutation frequency) and low mutation group (genomic stable group, 25% after mutation frequency). The ‘limma’ R package was used to analyse the difference in lncRNA expressions between the two groups, and the “survival,” “caret,” and “glmnet” R packages were used to screen lncRNAs that are related to clinical prognosis. Subsequently, a prognosis-related risk model was constructed and verified through different methods.

**Results:** According to the lncRNA expression data in TCGA, we found that seven lncRNAs (i.e. AL645608.6, LINC01436, AL645608.2, AC073534.2, LINC02593, AL512413.1, and AL645608.4) were highly correlated with the clinical prognosis of patients with AML, so we constructed a prognostic risk model of lncRNAs based on LINC01436, AC073534.2, and LINC02593. Gene Ontology and Kyoto Encyclopedia of Genes and Genomes pathway analyses of differentially expressed lncRNA-related target genes were performed, receiver operating characteristic (ROC) curves were created, the applicability of the model in children was assessed using the TARGET database and the model was externally verified using the GEO database. Furthermore, different expression patterns of lncRNAs were validated in various AML cell lines derived from Homo sapiens.

**Conclusions:** We have established a lncRNA prognostic model that can predict the survival of patients with AML. The Kaplan-Meier analysis showed that this model distinguished survival differences between patients with high- and low-risk status. The ROC analysis confirmed this finding and showed that the model had high prediction accuracy. The Kaplan-Meier analysis of the clinical subgroups showed that this model can predict prognosis independent of clinicopathological factors. Therefore, the proposed prognostic lncRNA risk model can be used as an independent biomarker of AML.

## 1 Introduction

Acute myeloid leukaemia (AML) is a malignant clonal disease of the haematopoietic stem and progenitor cells (Pelcovits and Niroula, 2013). In China, leukaemia affects 3–4 individuals per 100,000 populations. Among deaths due to malignant tumours, leukaemia ranked sixth in men, seventh in women and first in children and adults aged <35 years. In China, the incidence of acute leukaemia (AL) was significantly higher than that of chronic leukaemia, and AML was the most common (1.62/100,000). In recent years, intensive chemotherapy, haematopoietic stem cell transplantation and rigorous supportive treatment have greatly improved the prognosis of patients with AML aged <60 years. Moreover, 30% of patients with non-acute promyelocytic leukaemia are expected to survive for a long time (Sasine and Schiller, 2016). However, for these patients, a good quantitative model for predicting survival time is still not established (Song et al., 2018).

With the development of sequencing technology, the detection of leukaemia-related genes is becoming increasingly impeccable, which has increasingly attracted the attention of researchers. Among them, the long noncoding RNA (lncRNA) has gradually become a research hotspot. LncRNA is an RNA molecule with a length of >200 bp that originates from the noncoding region of the genome. It regulates gene expression at the transcriptional and post-transcriptional levels and participates in various biological functions (Al-Kershi et al., 2019). Some studies have shown that lncRNAs play an important role in many life activities, such as dose compensation effect, epigenetic regulation, cell cycle regulation and cell differentiation regulation (Peng et al., 2017). Recent studies have confirmed that changes in lncRNAs are related to the occurrence and development hematological malignancy, especially in AML. Myeloid-specific and polyadenylated lncRNA LOUP was found to induce myeloid differentiation and inhibits cell growth, acting as a transcriptional inducer of the myeloid master regulator. LOUP recruits RUNX1 to both the LOUP enhancer and the promoter, leading to the formation of an active chromatin loop (Trinh et al., 2021). Yin et al.(Yin et al., 2021) found that lncRNA DUBR highly expressed in AML, resulting in poor prognosis, especially in M4 AML. *In vitro* studies elucidated that knockdown of DUBR suppress the survival colony formation ability in AML cells. Academics pointed out that the activation of HOXBLINC, a HOXB locus-associated lncRNA, is a critical downstream mediator of NPM1c(+)-associated leukemic transcription program and leukemogenesis. HOXBLINC loss attenuates NPM1c(+)-driven leukemogenesis by rectifying the signature of NPM1c(+) leukemic transcription programs. Overexpression of HOXBLINC in mice enhances hematopoietic stem cell self-renewal and expands myelopoiesis, leading to the development of AML-like disease, reminiscent of the phenotypes seen in the Npm1 mutant knock-in (Npm1(c/+)) mice (Zhu et al., 1956). Gourvest et al.(Wei and Wang, 2017; Zhao et al., 2018; Liang et al., 2020; Gourvest et al., 2021) report an identification of lncRNA LONA overexpressed in NPM1-mutated AML patients. While NPM1 is nuclear and LONA cytoplasmic in wild-type NPM1 AML cells, LONA becomes nuclear as mutant NPM1 moves toward the cytoplasm. Gain or loss of function combined with a genome-wide RNA-seq identified a set of LONA mRNA targets encoding proteins involved in myeloid cell differentiation and interaction with its microenvironment. LONA overexpression exerts an anti-myeloid differentiation effect in mutant NPM1 established cell lines and primary AML cells. *In vivo*, LONA overexpression acts as an oncogenic lncRNA reducing the survival of mice transplanted with AML cells and rendering AML tumors more resistant to cytarabine chemotherapy.

This study aimed to combine the clinical data of AML from TCGA to obtain prognosis-related biomarkers, construct a prognostic risk model related to lncRNAs in AML and help patients with AML make clinical treatment decisions. In this study, transcription data of 151 patients with AML were downloaded from The Cancer Genome Atlas (TCGA). Perl language was used to collate the data, and R language was used for data analysis in an attempt to determine effective prognostic biomarkers for AML and construct a prognostic risk model using lncRNA in patients with AML.

## 2 Methods

### 2.1 Research Objects and Data Acquisition

Transcription group data (transcription profiling) and corresponding clinical data of 151 patients with AML were obtained through TCGA website (https://tcga-data.nci.nih.gov/tcga/). The database contains clinical data such as patient number, age, survival time and survival status. The genome mutation data (simple nucleotide variation) of 149 patients were also downloaded from TCGA.

### 2.2 Acquisition of the Expression Matrix of lncRNAs

On the official website of TCGA (https://gdc.nci.nih.gov/), the TCGA-LAML transcriptional group data (transcription profiling) were checked on the GDC Data Portal to download relevant raw htseq-count data, manifest and metadata files. In the CMD environment, the Perl language script was used to extract the original count data to form an expression matrix. The identification transformation of the transcription expression profile was implemented as Homo_sapiens.GRCh38.95. chr.gtf and downloaded from the Ensemble website, the gene expression profile matrix was obtained after comparison, and lncRNA was extracted using Perl language script to obtain the lncRNA expression matrix of patients with myeloid leukaemia.

### 2.3 Sample Mutation Frequency and Grouping

In the CMD environment, Perl language scripts were used to calculate the mutation frequency of the samples. In this study, 31 patients with the top 25% mutation frequency were assigned to the genomic instability group (high mutation group, genomic unstable [GU]) and 24 patients with the bottom 25% mutation frequency to the genomic stability group (low mutation group, genomic stable [GS]).

### 2.4 Screening of Differentially Expressed lncRNAs and mRNAs in the High and Low Mutation Groups and Gene Ontology and Kyoto Encyclopedia of Genes and Genomes Analyses

To determine potential AML biomarkers, R language was used to calculate the mean value of lncRNA in the GS and GU groups, and the “limma” R package was then used to set the threshold to logFC >1.0 and *p* < 0.05 to screen the differentially expressed IncRNAs between the two groups. Thereafter, data of the upregulated and downregulated differentially expressed IncRNAs and their corresponding expressions were saved, and the “pheatmap” R package was used to draw a heat map. We used the “limma” R package to test the correlation between the expressions of mRNA and lncRNA in the samples and obtained the correlation coefficient and *p* value. The mRNAs of the first 10 related genes were selected as the target genes of the corresponding differentially expressed lncRNAs. Subsequently, GO and KEGG enrichment analyses of the target genes were performed using “clusterProfiler” (version 3.14.3). The minimum and maximum genes were set to 5 and 5,000, respectively. A *p* value of <0.05 and a false discovery rate (FDR) of <0.25 were considered significant.

### 2.5 Cox Regression Analysis

To determine which of the differentially expressed genes were related to prognosis, we extracted the survival time and survival status of patients with AML and excluded data of patients whose survival time was less than 30 days. The survival time data were compared individually with differential gene expression data, and duplicated samples of expression data were removed; finally, 128 samples with complete prognosis were screened. Thereafter, the survival time data and differential gene expression data were combined into a matrix. Furthermore, we used the random function of R language to randomly divide the 128 samples into the train and test groups. First, we determined the prognosis-related lncRNAs from the train group for Cox analysis. Specifically, the univariate regression analysis was performed on the train group using the “survival” and “survminer” R packages. Seven lncRNAs related to prognosis were obtained, and the corresponding forest map was drawn by R packages “survival,” “caret,” “glmnet,” and “survminer.” Thereafter, the “glmnet” and “survival” R packages were used in the multivariate Cox regression analyses of the seven lncRNAs.

### 2.6 Construction of the lncRNA Prognostic Model

Using multivariate regression, a survival-related prognosis model was constructed based on three lncRNAs out of 7 that screened through multivariate regression. The model formula is as follows:

$$\mathrm{Risk}\;score={\Sigma^{3}}_{i=1}\beta i.$$

In this equation, **β** is the regression analysis coefficient of each IncRNA after multifactor Cox regression analysis, and **i** is the correlation of lncRNA of the multifactor regression. We defined the risk score of single samples lower than the median risk score of the training group as low risk and conversely as high risk. Accordingly, samples were divided into the high-risk subgroup and the low-risk subgroup in the train and test groups, respectively.

### 2.7 Visualization, Evaluation and Testing of the lncRNA Prognostic Model

#### 2.7.1 Testing the Clinical Characteristic Preference Between the Train Group and the Test Group Randomly Divided Into Subgroups

To test whether the clinical characteristics of patients with AML were consistent, patients with AML were divided into the elderly (≥60 years) and non-elderly (<60 years) groups and male and female groups. The chi-square test was used to process the percentage of age and sex in the two groups.

### 2.7.2 Survival Analysis Verification

To test the applicability of the prognostic model in the identification of patients with high- and low-risk status, we used the “survival” and “survminer” R packages to draw the high- and low-risk Kaplan–Meier curves of the train, test and overall groups.

#### 2.7.3 Receiver Operating Characteristic (ROC) Curve

To verify the accuracy of the model in predicting the prognosis of patients with AML in different periods, we used the “survival,” “survminer,” and “timeROC” R packages to draw the ROC curves of the 1-year and 5-year survival rates of the train, test and overall groups and calculated the area under their ROC curves (AUCs).

#### 2.7.4 Relationship Between Known Clinical Prognostic Genes and Gene Mutation Frequency and Prognostic Model

##### Relationship Between Patient Risk Score and Gene Expression and Between Patient Score and Genomic Instability

To examine the relationship between the prognostic model and other factors, we ranked the samples according to the risk score from low to high. Thereafter, we used the “limma” and “pheatmap” R packages to visualise the expression of three lncRNAs related to multivariate regression in the train, test and overall groups. Moreover, we visualised the mutation frequency of each sample and the known mutation-driven genes.

##### Relationship Between the Prognostic Model and Known Mutation-Driven Genes Such as TP53

We used the “limma” and “sparcl” R packages to extract the differentially expressed lncRNAs and mutation statistics of all samples. Thereafter, the samples were divided into GS (GS-like) and GU (GU-like) groups by cluster analysis. Then, the mutation frequency between the two types and the expression of the mutant gene LUNAR1 were evaluated, and the “ggpubr” R package was used to draw the corresponding box diagram. To examine the effect of common mutations on this prognostic model, we used the “survival” and “survminer” R packages to draw the Kaplan–Meier curves of the first six genes with the highest mutation frequency in the GS (GS-like) and GU (GU-like) groups. To test whether there was a significant difference in gene mutations between the high- and low-risk groups, we used “plyr” and “ggplot2” R packages to draw single mutation frequency histograms of common mutations in the train, test and overall groups to verify whether known common mutations affect the prognostic model of high- and low-risk scores.

#### 2.7.5 Verification of the Prognostic Model With Different Clinical Characteristics

To test the ability of the prognostic model to evaluate high- and low-risk groups with different clinical traits, we divided the samples into the elderly and non-elderly groups, male and female groups, non-M3 groups and high mutation frequency and low mutation frequency groups according to the clinical traits. Note that: 1. the survival analysis of M3 (acute promyelocytic leukaemia) group is not going to be performed as the sample size in this group is relatively small. 2. TCGA-AML data contains mutation data of 134 patients and complete clinical information 128 patients, taking the intersection of them, there are 88 patients with complete mutation data and clinical information. High mutation frequency was defined as single sample mutation counts ≥ median mutation counts, and low mutation frequency as single sample mutation counts < median mutation counts. Then, the “survival” and “survminer” R packages were used to draw the Kaplan–Meier curves of above-mentioned groups at high and low risks.

#### 2.7.6 Applicability of the Prognostic Model in Children

We downloaded data of 155 child patients with AML with complete prognosis-related lncRNA expression and clinical information from the TARGET database (https://ocg.cancer.gov/programs/target). Then, Microsoft Excel software was used to sort out the data into the children group and calculated the corresponding risk score. Finally, we used the “survival” and “survminer” R packages to draw the corresponding Kaplan–Meier curves between different groups of children and adults to test the applicability of the model in children.

#### 2.7.7 External Verification

GSE106291 (Herold et al., 2018) data from the GEO database, which included survival information and transcriptome data of 250 patients with AML, were downloaded for further analysis. As not every lncRNA was clearly annotated in this data (especially for RNA-seq data of early year), we calculated the risk score by determining the RNA-related expression level according to LNCipedia (Volders et al., 2019) and catRAPID (Armaos et al., 2021), in which different lncRNA transcripts were considered to belong to a certain gene if they share at least one (partially) overlapping exon and reside on the same DNA strand. In this way, transcripts were clustered into genes. The risk score was thus calculated according to the gene expression when lncRNA was not clearly annotated. Then, we used the “survival” and “survminer” packages for prognostic analysis. Finally, 123 patients with complete survival information and transcriptome data were included in the validation cohort.

#### 2.7.8 LncRNA Expression Verification in AML Cells

The LncRNAs were detected in AML cell lines derived from Homo sapiens with fluorescence *in situ* hybridisation (FISH) based on the protocol (Duncan et al., 2019). The methods were as follows: The specimens were permeabilised with cold 0.1% Triton X-100. The pre-hybridisation buffer was discarded, and hybridisation was performed using the LINC01436, AC073534.2 and LINC02593 probe overnight, respectively. After washing with SCC buffer, the coverslip was dyed with 4′,6-diamidino-2-phenylindole (DAPI), and the fluorescence test was conducted with laser scanning confocal microscope. AML cell lines were selected as follows: HL-60 (derived from the peripheral blood of patients with acute promyelocytic leukaemia), U937 (derived from the peripheral blood of patients with acute monocytic leukaemia), MV4-11 (derived from the peripheral blood of patients with biphenotypic B myelomonocytic leukaemia with FLT3-ITD mutation) and Kasumi-1 (derived from the peripheral blood of patients with acute myeloblastic leukaemia with AML1-ETO fusion gene positive). All cell lines were ATCC sources. Cells were maintained in RPMI-1640 medium supplemented with 10% foetal calf serum (HyClone Laboratories, Logan, UT, United States), 100 U/mL penicillin and 100 μg/ml streptomycin at 37°C in a humidified atmosphere of 5% CO_2_.

## 3 Statistical Analysis

Data analyses were performed in R language (R4.0.2), and the difference was considered significant when *p* < 0.05.

## 4 Results

### 4.1 Establishment of the lncRNA Prognostic Model for AML

#### 4.1.1 Differentially Expressed lncRNAs in TCGA Patients With AML

**FIGURE 1:**
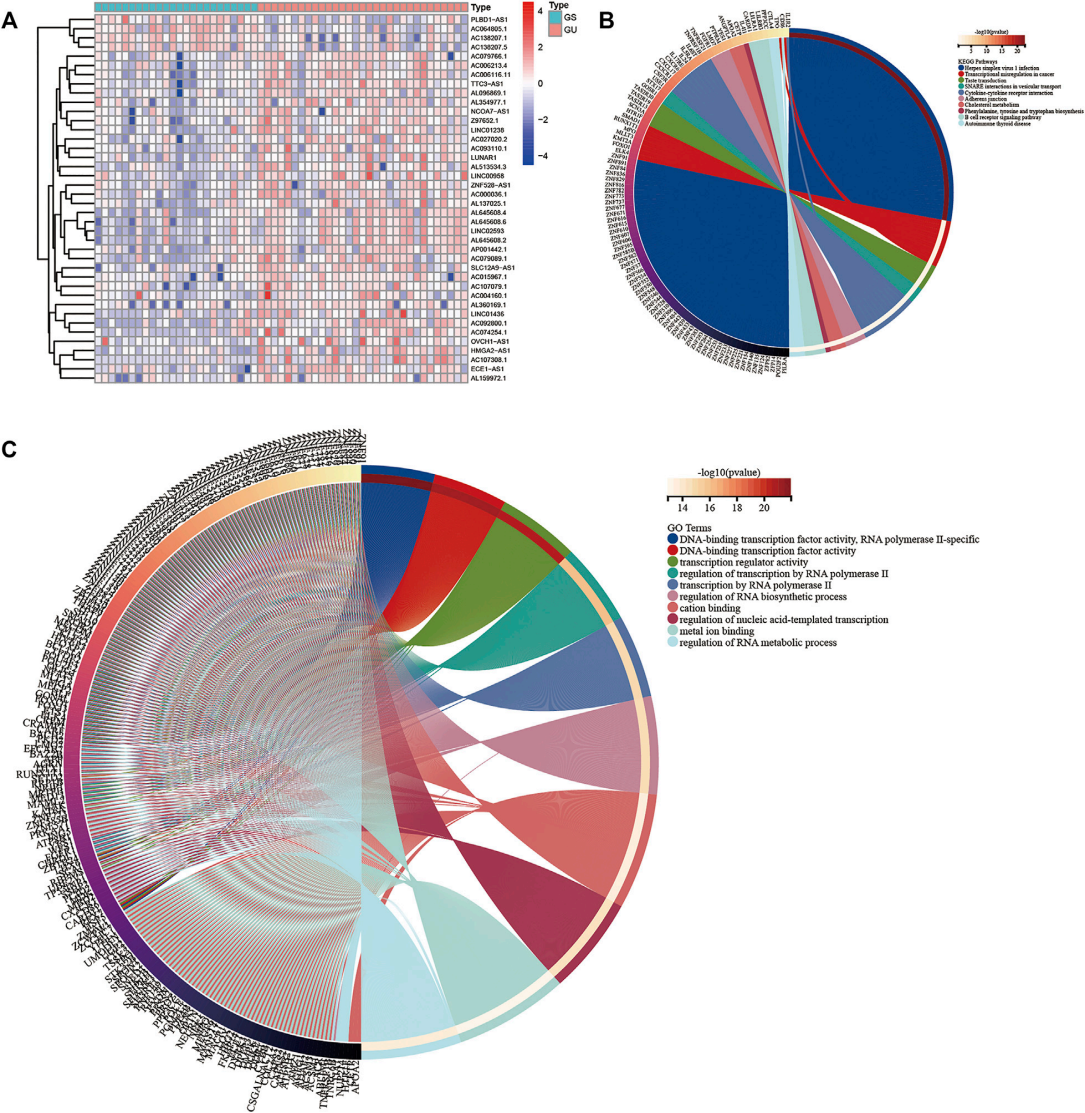
**(A)** Heat map of the top 20 upregulated and downregulated lncRNAs. The upregulated and downregulated genes are marked red and blue, respectively. The top 10 mRNAs related to differentially expressed lncRNAs were selected as target genes. The Kyoto Encyclopedia of Genes **(B)** and Genomes and Gene Ontology **(C)** analysis maps of the target genes were drawn.

We obtained 149 AML samples with transcriptome data from TCGA database. A total of 31 patients with the first 25% mutation frequency were classified as the GU group (high mutation group, mutCount ≥19), and 24 patients with post 25% mutation frequency were classified as the GS group (low mutation group, mutCount ≤3). The average expressions of lncRNAs in the GS and GU groups were calculated by R language, differentially expressed lncRNAs were screened using the “limma” R package and the threshold was set to (logFC >1.0 and *p* < 0.05). Finally, 59 differentially expressed lncRNAs were obtained (Supplementary Table S1). Among them, heat maps of 20 upregulated and 20 downregulated differentially expressed lncRNAs were drawn (Figure 1A). To further examine the human functions these lncRNAs are involved in, we performed KEGG and GO enrichment analyses of 59 target genes of differentially expressed lncRNAs. The results (Figures 1B,C) revealed that the most abundant genes in the KEGG were enriched in “Herpes simplex virus 1 infection” pathway. Regarding GO, the most enriched genes were in the ‘DNA-binding transcription factor activity, RNA polymerase II-specific’ process.

#### 4.1.2 lncRNA Prognostic Risk Model for AML

**FIGURE 2:**
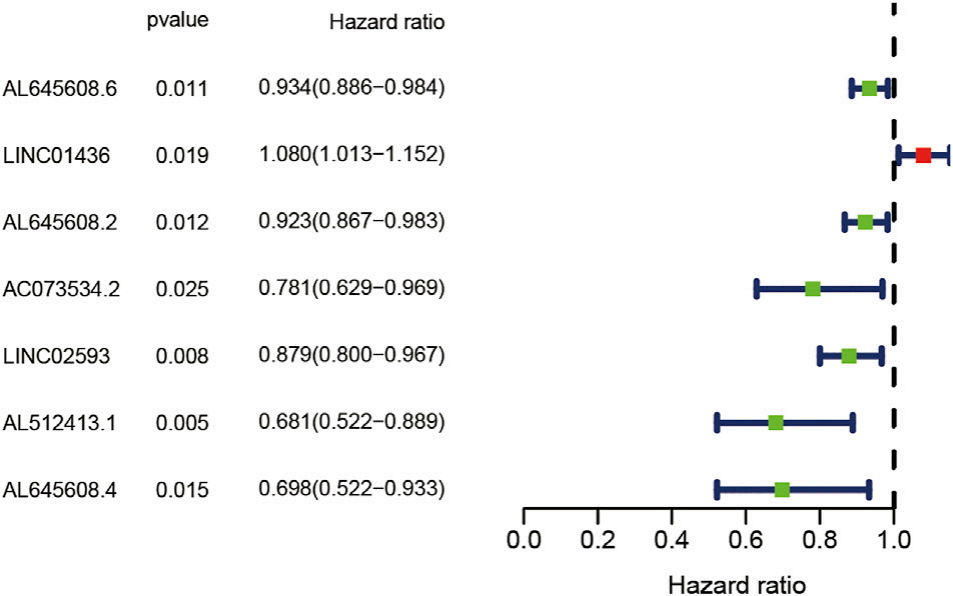
Forest plot of the univariate Cox regression analysis of seven lncRNAs with prognostic value. The *X*-axis represents the risk ratio, and the *Y*-axis represents the lncRNAs with prognostic value. Red represents a high-risk factor, which is negatively correlated with the survival time of the patient. Blue represents a low-risk factor, which is positively correlated with the survival time of the patient.

To evaluate the prognostic value of lncRNAs in AML, 128 samples were randomly divided into the train and test groups by R language random function. Thereafter, the differentially expressed lncRNAs in the train group were analysed by Cox regression analysis. Seven lncRNAs (AL645608.6, LINC01436, AL645608.2, AC073534.2, LINC02593, AL512413.1, and AL645608.4) related to prognosis were obtained, and their corresponding forest maps were drawn. As shown in Figure 2, among the seven lncRNAs, only the hazard ratio (HR) of LINC01436 is greater than 1, which means that LINC01436 is a risk factor for patients with AML and has a negative correlation with the clinical prognosis; therefore, the higher the expression, the worse the prognosis. The rest of the lncRNAs (AL645608.6 [HR = 0.934], AL645608.2 [HR = 0.923], AC073534.2 [HR = 0781], LINC02593 [HR = 0.879], AL512413.1 [HR = 0.681] and AL645608.4 [HR = 0698]) appeared as protective factors, which were positively correlated with the clinical prognosis of the patients, i.e. the higher the expression, the better the prognosis. Furthermore, we used “glmnet” and “survival” R packages to perform multivariate Cox regression analysis of the seven lncRNAs, and the key result of model construction was obtained. As shown in Supplementary Tables S2, S3 of 7 lncRNAs (LINC01436, AC073534.2 and LINC02593) were selected as major parameters to build the model, and they were identified as the independent prognostic factors through multivariate Cox regression analysis (coef [LINC01436] = 0.070402, coef [AC073534.2] = −0.303302254, coef [LINC02593] = −0.139241309). The risk score of each patient could be calculated according to the regression coefficient and expression value of the three lncRNAs.

### 4.2 Visualization, Evaluation, and Testing of the lncRNA Prognostic Model

#### 4.2.1 Analysis of the Clinical Characteristic Preference Among Random Groups

To determine the consistency of the clinical characteristics during model construction, clinical features of all samples were compared by the chi-square test. As shown in Supplementary Table S3, the *p* value between the train and test groups were all >0.05, indicating that our model grouping has no characteristic preference.

#### 4.2.2 Clinical Grouping Verification of the Prognostic Model

**FIGURE 3:**
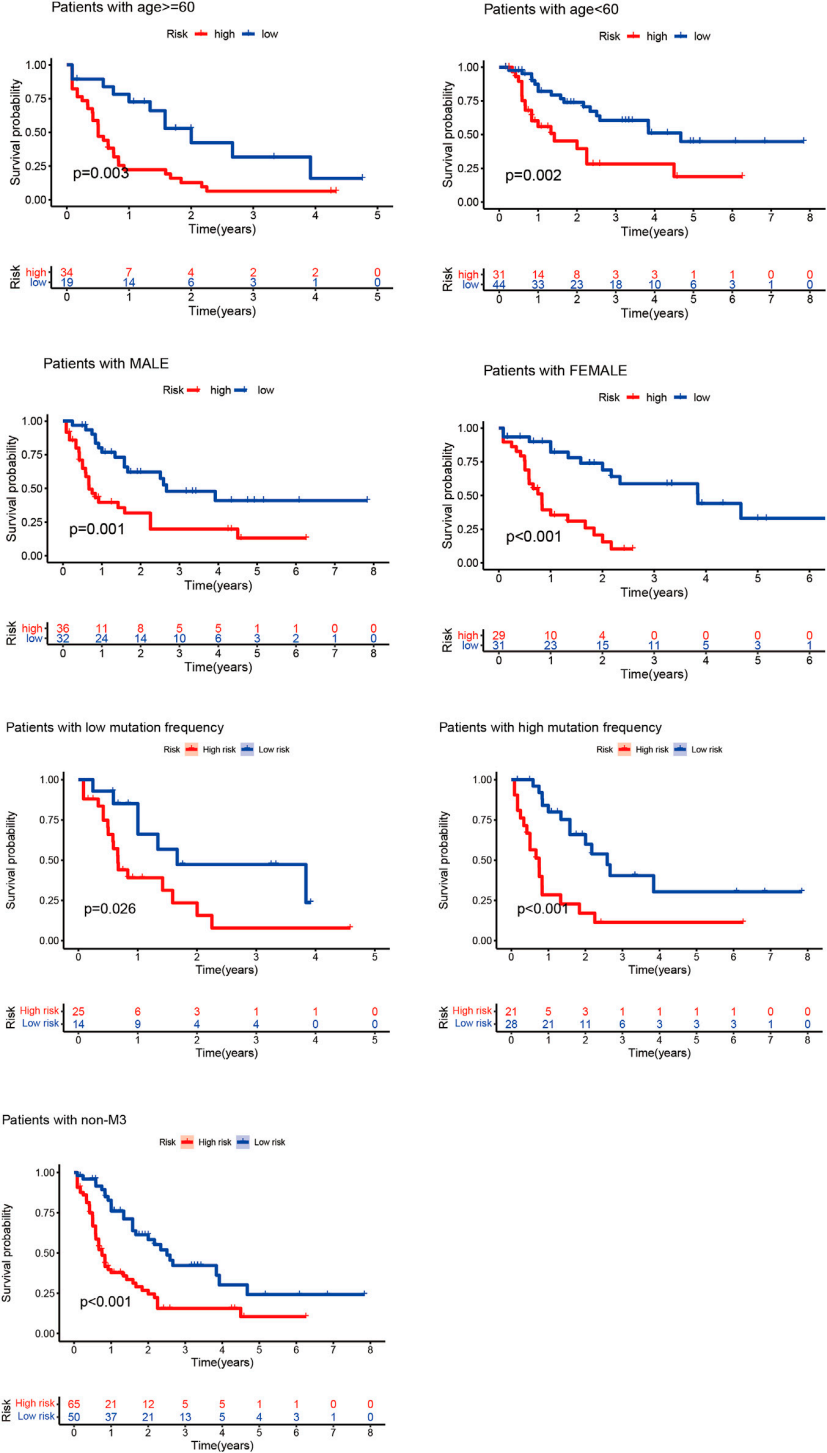
Kaplan–Meier curves of high- and low-risk groups of elderly and non-elderly patients and male and female patients. In the Kaplan–Meier curve of different clinical subgroups of patients with AML, the X- and *Y*-axes represent the time and probability of survival, whereas the red and blue lines represent the high- and low-risk groups, respectively.

According to the clinical characteristics, patients were divided into the elderly and non-elderly groups and male and female groups. The “survival” and “survminer” R packages were used to draw the Kaplan–Meier curves of the high- and low-risk patients in the elderly and non-elderly groups and male and female groups. As presented in Figure 3, the Kaplan–Meier curve showed that the survival time of patients in the low-risk group was significantly prolonged, and the median survival time in the non-elderly group was 2.17 years, which was higher than that in the high-risk group (0.84 years). In the elderly group, the median survival time in the low-risk group (1.58 years) was higher than that in the high-risk group (0.50 years). In the male group, the median survival time of the low-risk group (1.84 years) was higher than that of the high-risk group (0.63 years). In the female group, the median survival time of the low-risk group (1.84 years) was higher than that of the high-risk group (0.75 years). In the low mutation frequency group, the median survival time of the low-risk group (1.17 years) was higher than that of the high-risk group (0.66 years). In the high mutation frequency group, the median survival time of the low-risk group (1.66 years) was higher than that of the high-risk group (0.67 years). In the non-M3 group, the median survival time of the low-risk group (1.71 years) was higher than that of the high-risk group (0.67 years). These results showed that the prognostic model was not affected by the gender, age, FAB subtypes and mutation frequency of the patients, and the lncRNA prognostic prediction model demonstrated good applicability when patients were divided into high- and low-risk groups according to the clinicopathological characteristics, suggesting that the model is an independent index for predicting the prognosis of patients with AML.

#### 4.2.3 Verification of Survival Prediction

**FIGURE 4:**
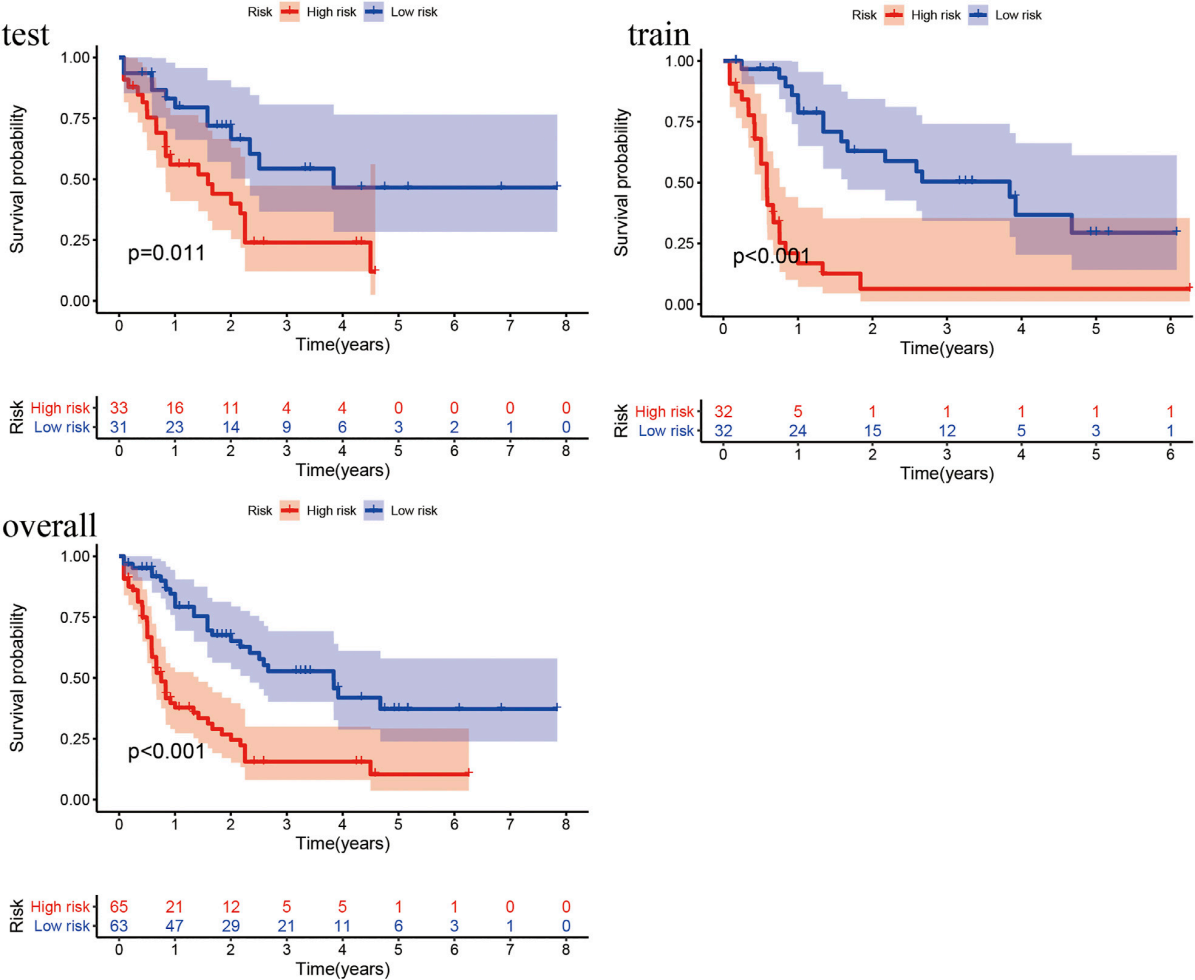
Kaplan–Meier curves of high- and low-risk groups in the train, test, and overall groups. The X- and *Y*-axes represent the time and probability of survival, whereas the red and blue lines represent the high- and low-risk groups, respectively.

According to the prognostic model, we constructed the high- and low-risk Kaplan–Meier curves of the train, test and overall groups and calculated the *p* value of the high and low survival curves. The median survival times were 1.84 and 0.67 years in the low- and high-risk groups of the overall group (Figure 4), 1.715 and 0.58 years in the train group, and 1.92 and 0.92 years in the test group, respectively. These results revealed that the survival rate of the low-risk group was higher than that of the high-risk group, indicating that the lncRNA prognostic prediction model showed good applicability for the survival prediction of patients with AML.

#### 4.2.4 ROC Curve

**FIGURE 5:**
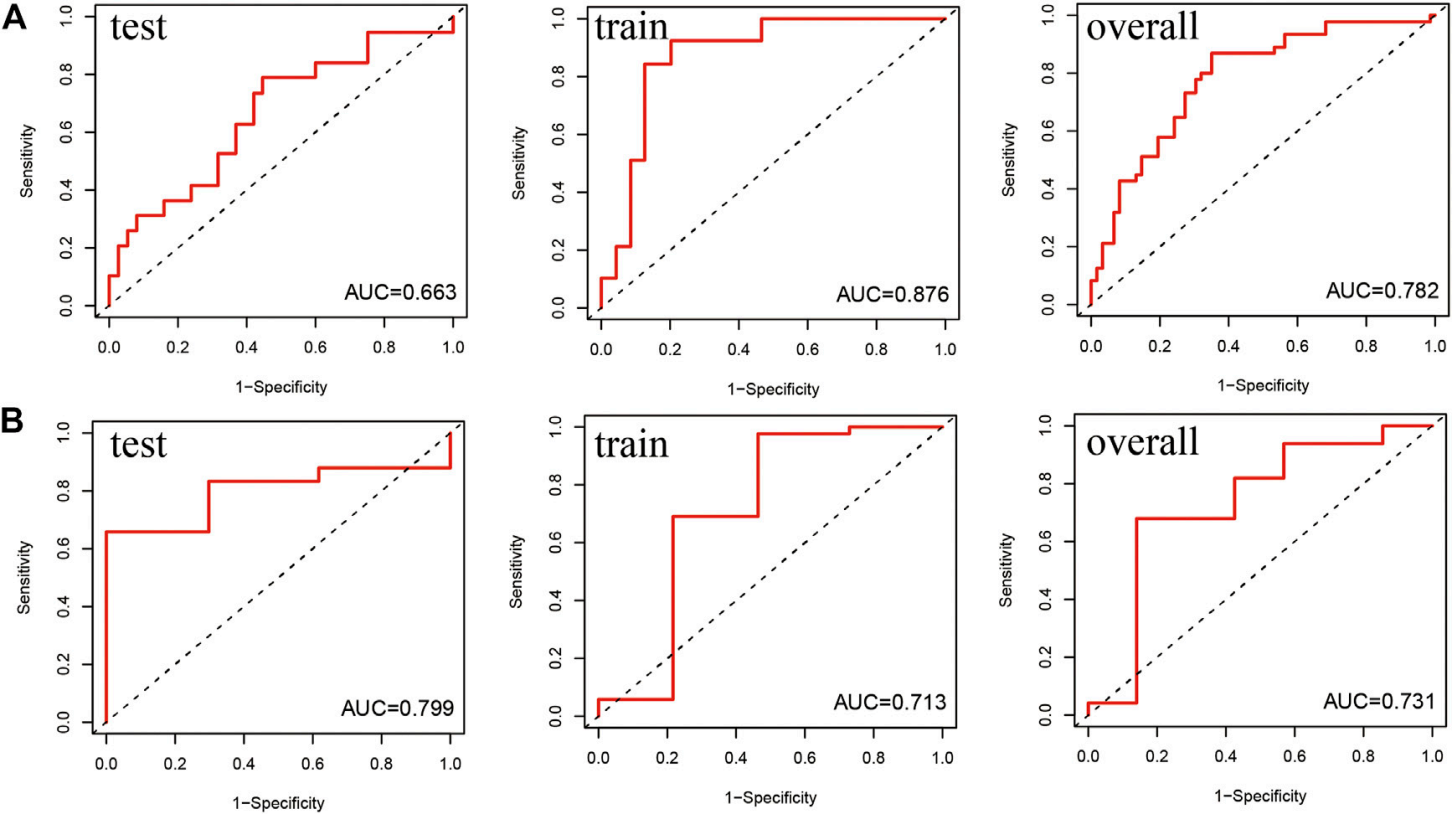
Receiver operating characteristic (ROC) curve of the prediction efficiency of the train, test, and overall groups. The X- and *Y*-axes represent 1-specificity and sensitivity, respectively. Corresponding ROC curve of the **(A)** 1-year survival rate and **(B)** 5-year survival rate.

To analyse the prognostic model, we constructed the ROC curves (Figure 5) of the 1-year and 5-year survival rates in the train, test and overall groups. As shown in Figure 5, the AUCs of the 1-year and 5-year survival rates were 0.876 and 0.713 in the train group, 0.663 and 0.799 in the test group, and 0.782 and 0.731 in the overall group, respectively. The results revealed that the lncRNA model had good prediction accuracy within 1–5 years and can predict the survival of patients with AML in other independent cohorts, and the accuracy of this model in predicting the 1-year survival rate (AUC = 0.782) was better than that of the 5-year survival rate (AUC = 0.731). The *p* values of the above-mentioned analyses were all less than 0.05.

#### 4.2.5 Relationship Between the Prognostic Model and Clinically Known Prognostic Genes

##### Relationship Between Sample Risk and Gene Expression, Patient Risk and Genomic Instability

**FIGURE 6:**
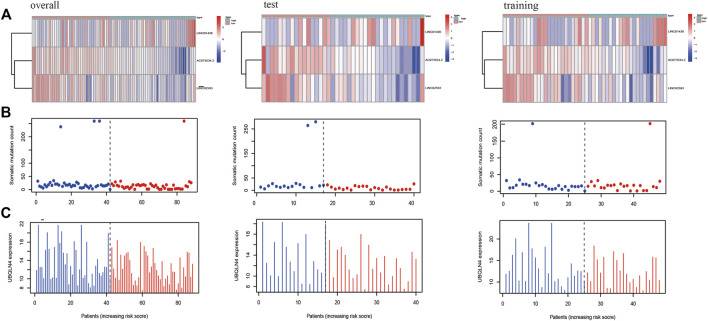
The *X*-axes of A, B, and C are all samples sorted according to the increasing values at risk. **(A)** Heat map of the lncRNAs related to the prognostic model; the ordinate represents the three lncRNAs that make up the prognostic model. Red represents upregulation of gene expression, while blue represents downregulation of gene expression. **(B)** Distribution of gene mutation frequency; the ordinate represents the mutation frequency of each sample. **(C)** Expression frequency distribution of UBQLN4, the driver of genomic instability; the ordinate is the expression of UBQLN4 in each sample.

To evaluate the relationship between predictive model scores and gene expression, gene mutation frequency, and mutation-driven genes, we ranked the samples of the train, test and overall groups according to the risk scores from low to high (from left to right). Thereafter, the expression heat maps, gene mutation frequency distribution maps and box maps of related genes of the three lncRNAs in the prognostic model were drawn. As shown in Figure 6, the abscissa presents all samples sorted according to the increasing risk prediction value of the model. The expressions of AC073534.2 and LINC02593 decreased gradually with the increase in the risk score (Figure 6A). The median frequency of gene mutation in the low-risk group (i.e. 16) was higher than that in the high-risk group (i.e. 12) (Figure 6B). The median frequency of gene mutation in the low-risk group was higher than that in the high-risk group. The median UBQLN4 expression of the genes driven by genomic instability in the low-risk group was 12.39 (mean, 13.12), whereas that of the high-risk group was 11.85 (mean, 12.16) (Figure 6C). From Figure 6, we can conclude that the frequency of the gene mutation and genomic instability are negatively correlated with the risk score of the patients.

##### Relationship Between the Prognostic Model and Known Mutant Genes Such as TP53

**FIGURE 7:**
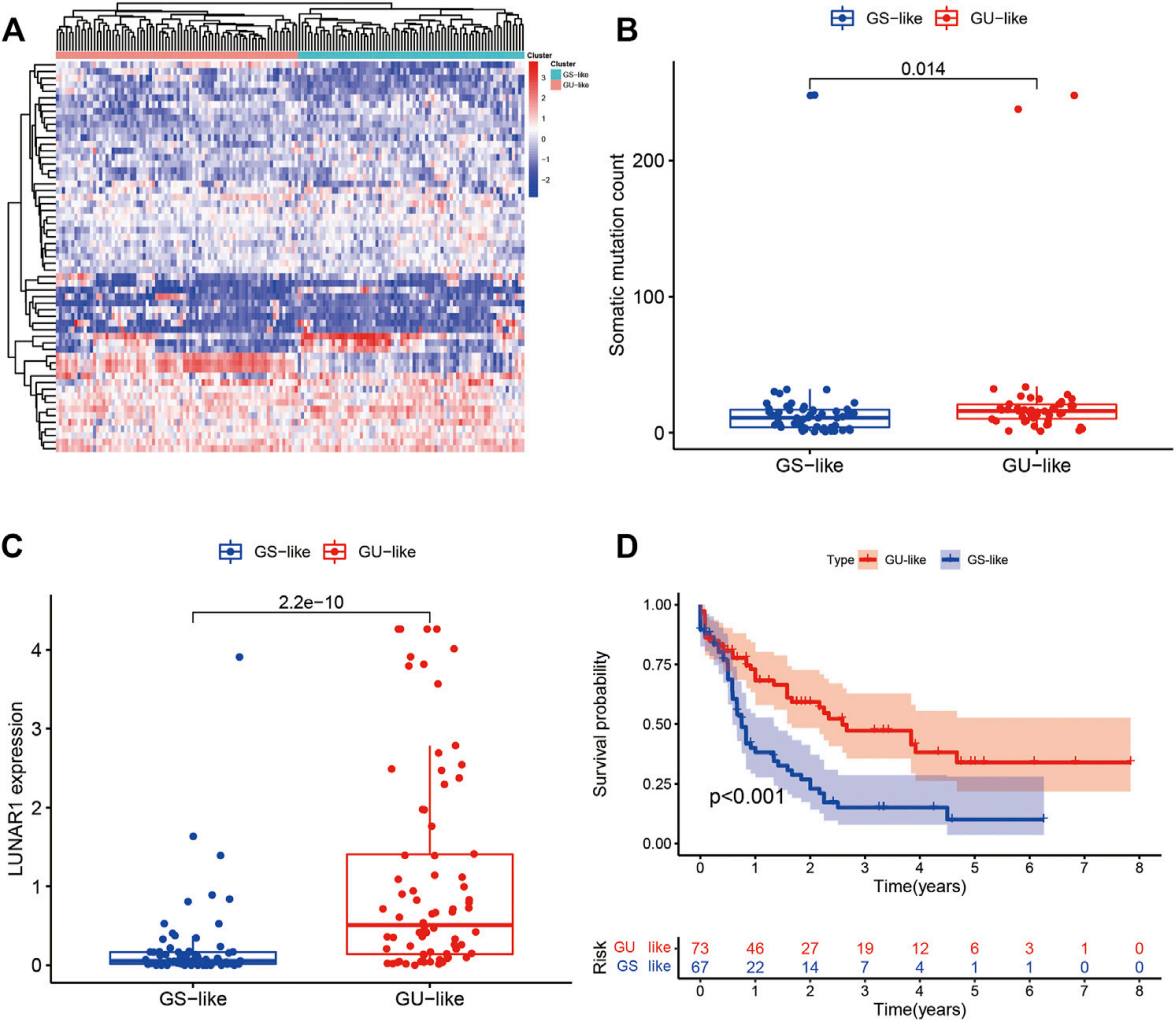
**(A)** Heat map of all samples after the cluster analysis. The *X*-axis is the sample type, blue represents the genomic stable type and red represents the genomic unstable type. The *Y*-axis represents differentially expressed lncRNAs, red represents upregulation, and blue represents downregulation. **(B)** Box diagram of the mutation frequencies of the two types. **(C)** Expression map of the two types of LUNAR1. **(D)** Kaplan–Meier curves of the GU-like and GS-like groups. In the Kaplan–Meier curve of different somatic mutation count of patients with AML, the X- and *Y*-axes represent the time and probability of survival, whereas the red and blue lines represent the GU-like and GS-like groups, respectively.

**FIGURE 8:**
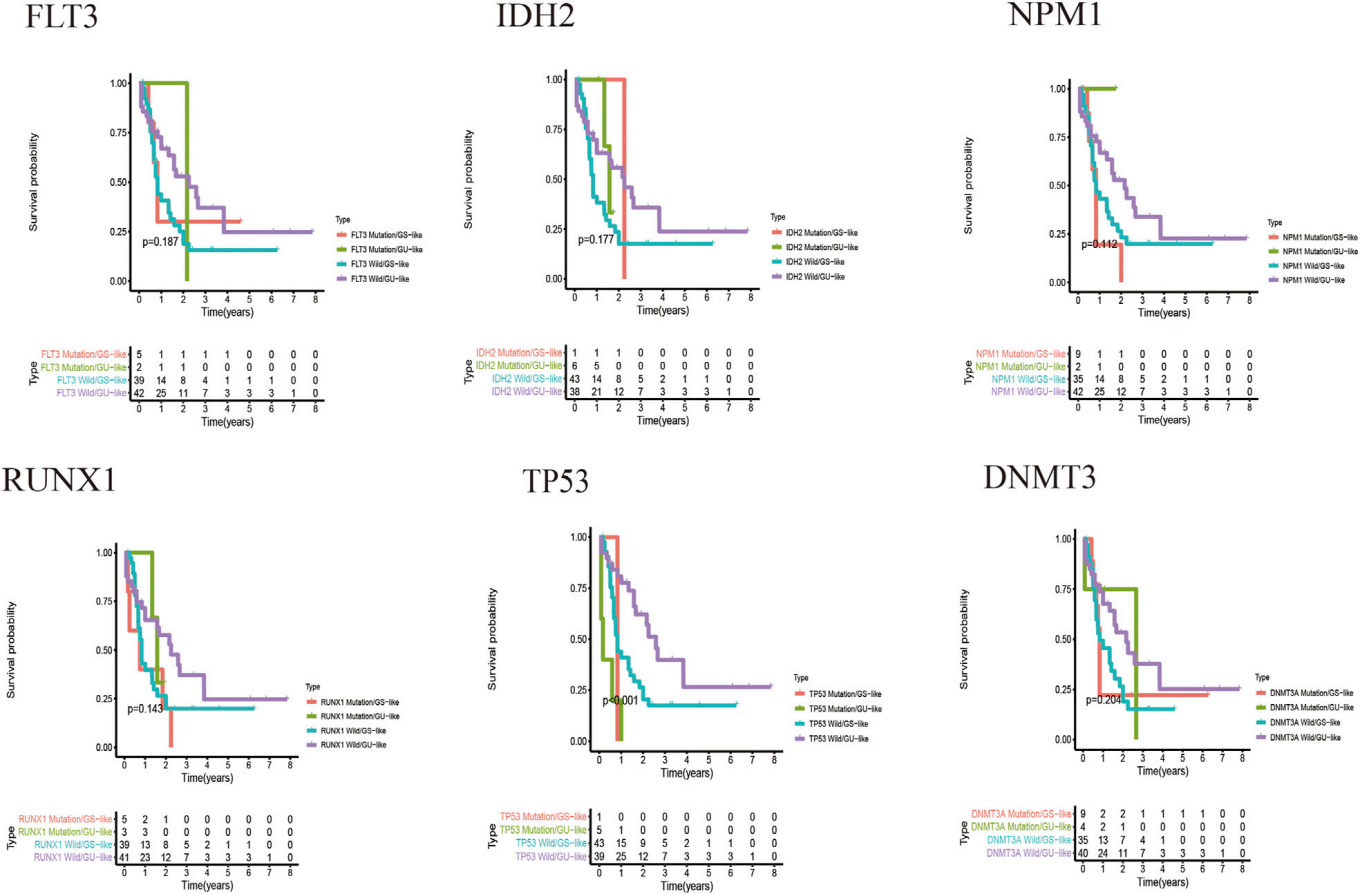
Kaplan–Meier curves of the genomic stable type and genomic unstable type under different gene mutations. The *X*-axis represents time, and the *Y*-axis represents survival probability.

**FIGURE 9:**
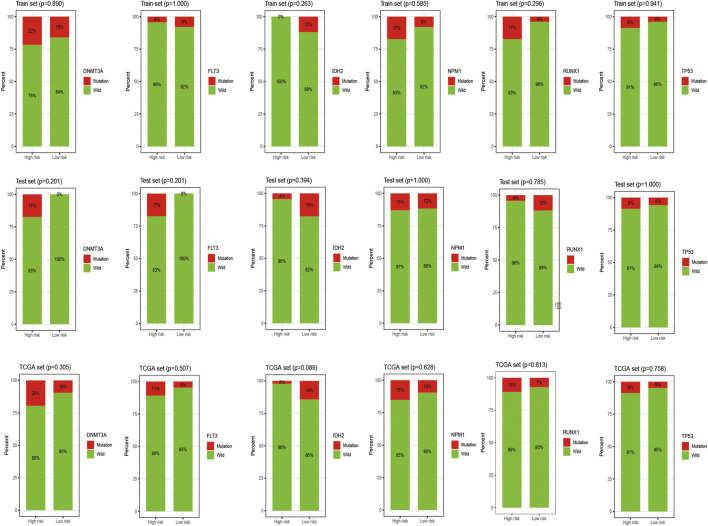
Histogram of the mutation frequency of common mutation genes in the train, test and overall groups. The *X*-axis represents the high- and low-risk groups, and the red on the *Y*-axis represents the ratio of samples with corresponding gene mutations.

Figure 7 shows the heat map after the cluster analysis. Figure 7A presents the lncRNA expression heat map of the GS type (GS-like) and GU type (GU-like). Figure 7B presents a significant difference in the mutation frequency between the two groups (*p* < 0.014). A significant difference was found in the expression of the visible gene *LUNAR1* (*p* < 0.05) (Figure 7C). As presented in Figure 7D, the Kaplan–Meier curve showed that the survival time of patients in the GU-like group was significantly prolonged, with a median survival time of 1.340 years, which was higher than that in the GS-like group (0.666 years). This means that patients with higher mutation frequencies have better overall survival (OS). Thereafter, the Kaplan–Meier curves of different gene mutations in the GS (GS-like) and GU (GU-like) groups were drawn. In Figure 8, among the first six genes with the highest mutation frequency, only the unstable gene group and the mutant TP53-positive group had a synergistic effect on the survival curve (*p* < 0.001). The specific reason is not clear, which may be related to the chemical resistance and high risk of recurrence of TP53 mutation (Döhner et al., 2010; Stengel et al., 2017; Welch, 2018; Barbosa et al., 2019). The effect of other gene mutations on the survival rate of gene instability had no clinical significance. Thereafter, we drew the bar chart of the mutation frequency of common genes in the train, test and overall groups. Figure 9 shows no significant difference in the expressions of mutant *DNMT3A*, *FLT3*, *IDH2*, *NPM1*, *RUNX1,* and *TP53* among the groups, indicating that the known prognostic genes do not affect the prediction of high and low risk in the prognostic model and that the risk score can be used as a prediction tool independent of the current prognostic-related genes.

### 4.3 Applicability of the Model in Child Patients

**FIGURE 10:**
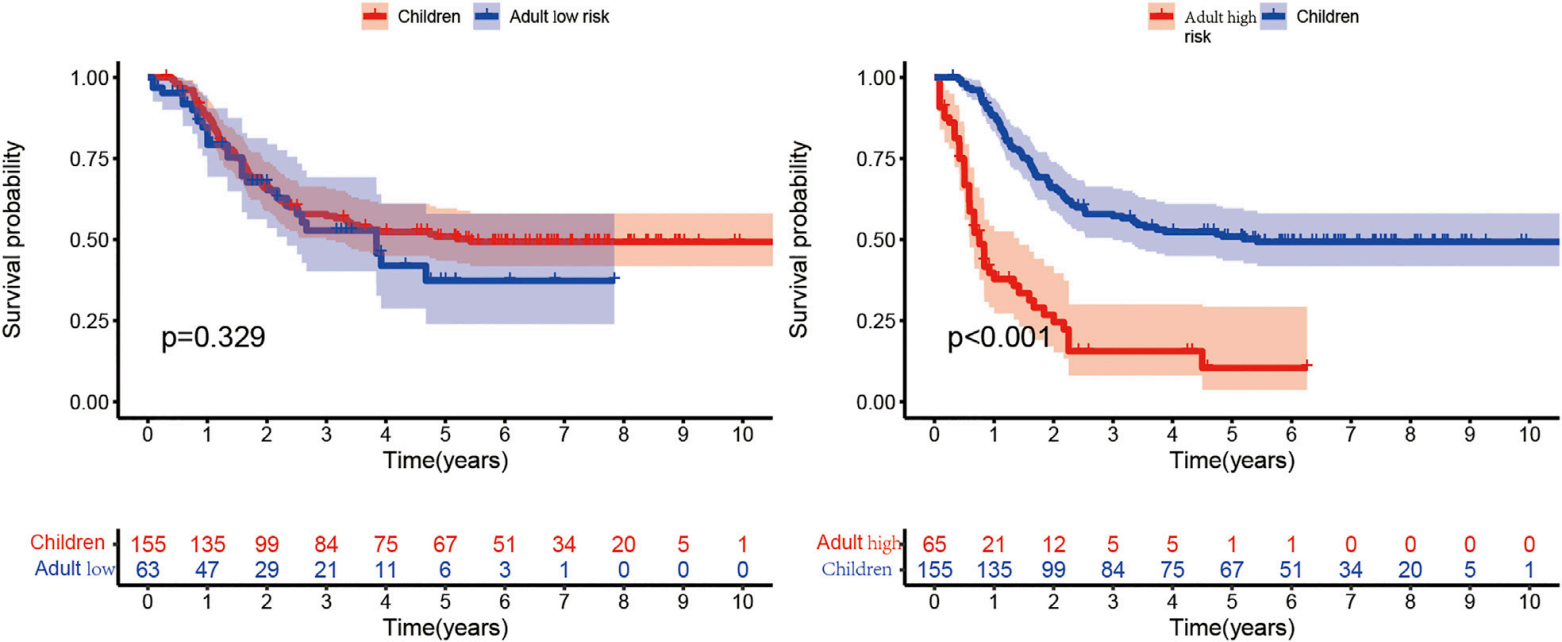
Comparison of the children group from the TARGET database with the high- and low-risk subgroups of the adult group. In the Kaplan–Meier curve, the X- and *Y*-axes represent the time and probability of survival, respectively.

The clinical characteristics of child samples from the TARGET database are summarized in Table 1. The Kaplan–Meier curve in Figure 10 shows that the median survival time of children is 3.67 years, which is higher than that of adults. In conclusion, the prognosis of the children group was significantly better than that of the adult high-risk group, and no significant difference was found between the children and adult low-risk groups. This finding suggested that the prediction model was not suitable for disease prediction and evaluation in children.

**TABLE 1:** Clinical characteristics of patients from the TARGET database included in the validation study.

	Children (n = 155)
Sex	
Male	79
Female	76
NPM mutation	
Yes	7
No	143
FLT3 PM	
Yes	11
No	144
FLT3/ITD positive?	
Yes	13
No	142
CNS disease	
Yes	10
No	145
Life status	
Alive	79
Dead	76

Abbreviation: PM, point mutation; CNS, central nervous system

### 4.4 External Verification

**FIGURE 11:**
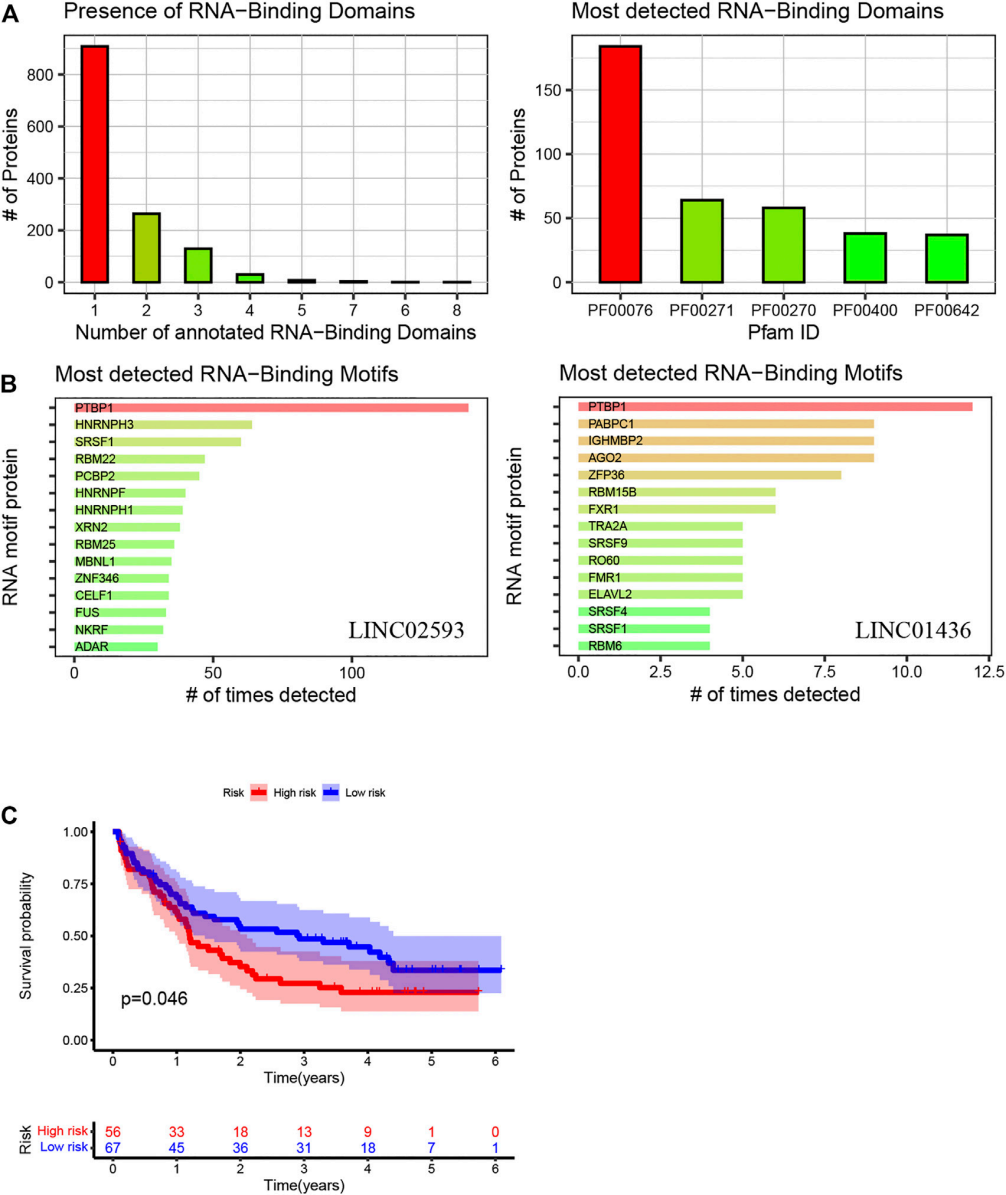
Using the method of LNCipedia (Volders et al., 2019) and catRAPID (Armaos et al., 2021), transcripts are clustered into genes. **(A,B)** indicate the presence of protein nucleic-acid-binding domains or RNA recognition motifs in LINC01436 and LINC02593. **(C)** Kaplan–Meier curves of high- and low-risk groups in the validation cohort. The X- and *Y*-axes represent the time and probability of survival, whereas the red and blue lines represent the high- and low-risk groups, respectively.

Patients with relatively short survival time (OS ≤ 30 days) were excluded. As mentioned in 4.2.5.2 of the Results section, high mutant TP53 expression could most independently affect OS. Patients with high TP53 mutation expression were also removed from the analysis. Finally, we performed a prognostic analysis in the validation cohort, which consisted of 123 samples with complete survival information and transcriptome data. The clinical characteristics are summarized in Table 2. The model was applied to stratify these patients into low-risk and high-risk groups. The median survival times of the low- and high-risk groups were 2.89 and 1.19 years, respectively, which indicated that the validation efficiency of the model in an external cohort is acceptable (Figure 11).

**TABLE 2:** Clinical characteristics of patients from GSE106291 included in the validation study.

	High risk (n = 56)	Low risk (n = 67)	P
Age			0.908562
≥60	27	33	
<60	29	34	
Sex			0.192443
Male	26	39	
Female	30	28	
RUNX1_mutation			0.642099
Yes	11	11	
No	45	56	
RUNX1-RUNX1T1_fusion			0.476547
Yes	1	4	
No	55	63	
Treatment response			0.641233
resistant	19	20	
sensitive	34	43	
Life status			0.115523
Alive	15	27	
Dead	41	40	

Abbreviation: RUNX1, runt-related transcription factor 1; RUNX1T1, rUNX1 partner transcriptional co-repressor 1. Significant P value is in bold typeface.

### 4.5 LncRNA Expression Pattern Verification in AML Cell Lines

**FIGURE 12:**
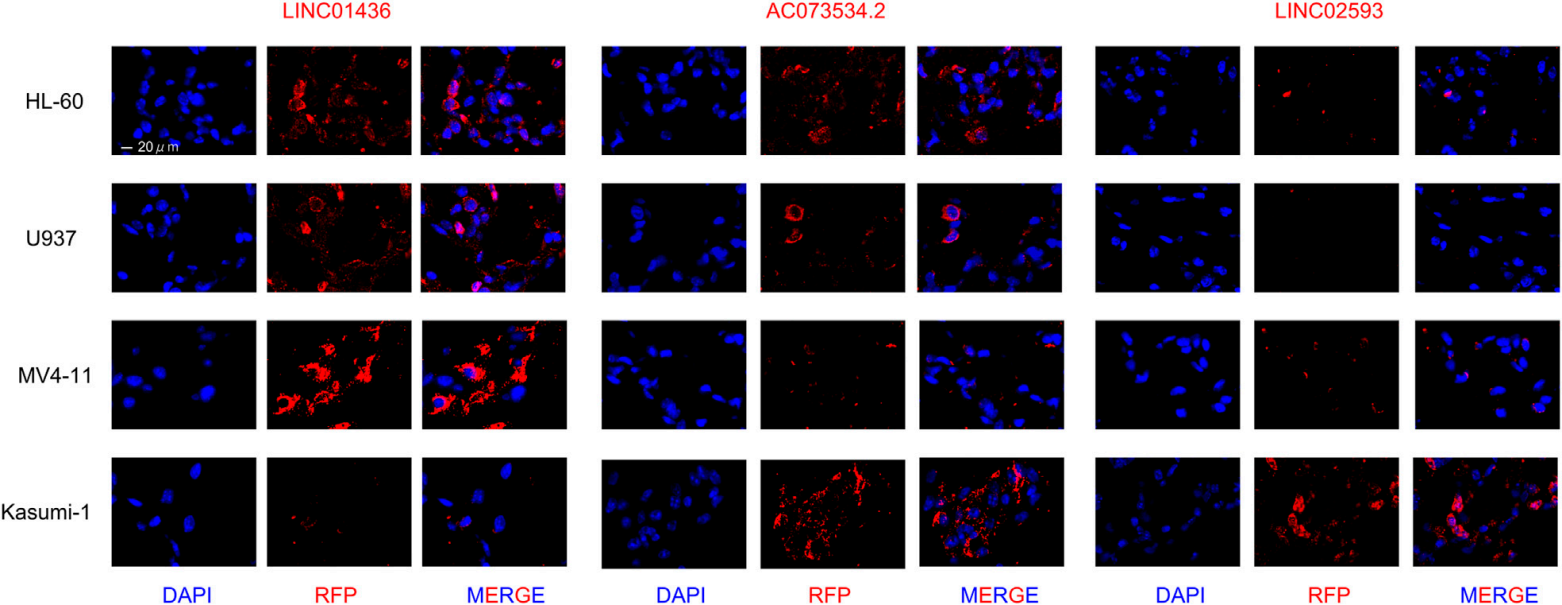
FISH staining in LINC01436, AC073534.2, and LINC02593 in AML cell lines HL-60, U937, MV4-11, and Kasumi-1 (original magnification ×630).

To determine the expression patterns of LINC01436, AC073534.2 and LINC02593 in various AML cells, we performed a FISH assay in four AML cell lines that may represent common clinical conditions. The results showed that LINC01436 was more expressed in MV4-11, but less expressed in Kasumi-1, AC073534.2 was significantly reduced in THP-1 and MV4-11 cells and LINC02593 was less found in all cell lines, but in Kasumi-1 (Figure 12). FLT3-ITD mutation is correlated with poor prognosis, and *AML1–ETO* fusion gene is associated with good survival in clinical practice. The above expression pattern of lncRNA may indicate their role in prognosis.

## 5 Discussion

AML is a serious threat to human health. However, a quantitative index to predict the prognosis of AML is still lacking. Previous studies on AML have mainly focused on mutant genes, namely, *FLT3*, *IDH2*, *NPM1*, *RUNX1*, *TP53,* and *DNMT3A* (Ofran, 2014; Sudhindra and Smith, 2014; Ohgami et al., 2015; Papaemmanuil et al., 2016), miRNA (Ali Syeda et al., 2020) and mRNA (Blagden and Willis, 2011). Researchers have extensively investigated the expression patterns of mRNAs and miRNAs, and many mRNAs or miRNAs have been identified as prognostic markers for patients with AML (Ge et al., 2019; Ibraheem et al., 2019; Elsayed et al., 2020). In recent years, a new class of noncoding RNA (lncRNA) has gradually become a research hotspot in various cancer fields (Ferrè et al., 2016; Paraskevopoulou and Hatzigeorgiou, 2016; Qian et al., 2019). RNA, which lacks protein-coding ability, is defined as noncoding RNA, which accounts for >98% of the human gene sequence. Approximately 90% of the noncoding sequences are transcribed, producing numerous noncoding transcripts, in which RNA with a length of more than 200 nucleotides is also known as lncRNA (Jathar et al., 2017). Some studies have reported the abnormal expression of lncRNAs in the occurrence and development of AML and found that some lncRNAs could be highly related to the prognosis (Garzon et al., 2014). Although some previous studies have confirmed a series of differences in the expression of lncRNAs in AML, the research on the value of lncRNA in predicting the clinical prognosis of AML is still limited. Except for our study, according to the expression and mutation degrees of *FLT3*, *DNMT3A*, *TP53* and other genes and chromosome changes, cases are classified as low, middle and high types; as a result, the survival time of patients was roughly estimated (Infante et al., 2018). However, a good quantitative model to analyse the survival of patients has not yet been established. Therefore, this study attempted to construct a prognostic risk model of lncRNA in patients with AML, to determine a potentially clinically applicable lncRNA prognostic model and to examine its high repeatability and practicability in different clinical groups.

Based on the mutation data of 149 samples, we used R language to divide the patients into a high and a low mutation group. Thereafter, differential lncRNA expressions of the high and low mutation groups were screened. We screened out seven lncRNAs related to prognosis, namely, AL645608.6, LINC01436, AL645608.2, AC073534.2, LINC02593, AL512413.1, and AL645608.4, using univariate Cox regression analysis and established the prognostic risk model formula based on multivariate Cox regression. Among them, the regression coefficient of LINC01436 was greater than 0, which was negatively correlated with the survival time, whereas the regression coefficients of two lncRNAs (AC073534.2 and LINC02593) were less than 0, which were positively correlated with the survival time. We extracted the regression coefficients of lncRNAs through multivariate Cox analysis and constructed three prognostic risk models composed of lncRNAs. Of the three lncRNAs, LINC01436 has been reported in gastric cancer, lung cancer and other diseases (Yuan et al., 2019; Lu et al., 2020; Xu et al., 2020; Zhang et al., 2020). However, no studies have investigated AC073534.2 and LINC02593. Furthermore, we used the prognostic model to calculate the risk score of each sample according to the median risk score. The sample was divided into high- and low-risk groups. We used R language to draw relevant heat maps, ROC curves and Kaplan–Meier curves. The ROC curve showed that the prognostic model was stable for predicting the 1-year and 5-year survival of patients with AML, indicating that the model has a good predictive ability. Moreover, under different clinical characteristics, the OS rate of the high-risk group was significantly lower than that of the low-risk group, indicating that the prognostic model can distinguish patients with different prognoses. Therefore, we are certain that the prognostic model can be used as an independent prognostic marker with a high clinical value.

However, this study has some limitations. First, our AML sample size and clinical data are limited. Second, our research results are preliminary, mainly based on a previously published dataset for secondary mining and analysis and thus lacks functional verification of lncRNAs. Therefore, further prospective studies are needed to verify our findings.

In conclusion, we have developed a lncRNA prognostic model that is significantly related to the prognosis of patients with AML. This model can accurately stratify patients and help determine whether patients are more active in treatment. Moreover, the predictive ability of the prognostic model is not influenced by clinicopathological factors such as age and sex; therefore, it has good applicability. Compared with the known prognostic biomarkers, the developed model is more convenient and intuitive in predicting the prognosis of patients with AML. If our prognosis model can be combined with the known biomarkers of AML molecules like FLT3-ITD, C-KIT mutation, et al. we can further screen high-risk groups and guide the clinical formulation of individualised treatment plans. Therefore, we believe that the prognostic model has a wide application prospect.

## Data Availability

The original contributions presented in the study are included in the article/Supplementary Material, further inquiries can be directed to the corresponding authors.
