# Supplementary material for: DesA Prognostic Risk Model of LncRNAs in Patients With Acute Myeloid Leukaemia Based on TCGA Data
Source: Front Bioeng Biotechnol. 2022 Feb 21;10:818905. doi: 10.3389/fbioe.2022.818905 (PMC8899517; doi:10.3389/fbioe.2022.818905)
Supplement: Supplementary file 1 [file Table1.docx]

**Supplementary Table-1：**the 59 differentially expressed lncRNAs between GS and GU group

| lncRNA | conMean | treatMean | logFC | pValue | fdr |
| --- | --- | --- | --- | --- | --- |
| LUNAR1 | 0.152689 | 0.951193 | 2.639146 | 0.00195 | 0.048778 |
| AC006116.11 | 0.683868 | 1.475307 | 1.109224 | 2.74E-05 | 0.015526 |
| AC006042.3 | 0.243562 | 0.577169 | 1.244702 | 8.93E-05 | 0.025301 |
| AL645608.6 | 1.665211 | 6.86567 | 2.043696 | 6.42E-05 | 0.023112 |
| AC010300.1 | 0.420018 | 1.239624 | 1.561381 | 0.000211 | 0.02988 |
| AC064805.1 | 4.022864 | 1.046311 | -1.94291 | 0.001461 | 0.046041 |
| AL359643.2 | 0.271862 | 0.665384 | 1.291312 | 1.60E-05 | 0.015526 |
| AL354977.1 | 0.69085 | 1.510945 | 1.129008 | 0.001297 | 0.046041 |
| AL513534.3 | 0.446525 | 1.325613 | 1.569845 | 0.000581 | 0.039751 |
| LINC01436 | 0.2414 | 5.864192 | 4.602434 | 0.001721 | 0.047723 |
| ECE1-AS1 | 0.289513 | 0.625802 | 1.112078 | 0.001139 | 0.046041 |
| AP001442.1 | 0.506786 | 1.598572 | 1.657336 | 0.000503 | 0.039751 |
| AC000036.1 | 0.199079 | 0.733947 | 1.882334 | 0.001549 | 0.046732 |
| AL117336.1 | 0.434108 | 1.046866 | 1.269951 | 0.000284 | 0.033712 |
| AL121929.3 | 1.149406 | 2.962967 | 1.366154 | 0.000306 | 0.033712 |
| PLBD1-AS1 | 0.954618 | 0.284398 | -1.74701 | 0.001158 | 0.046041 |
| AC079089.1 | 0.198195 | 0.92655 | 2.224949 | 0.001195 | 0.046041 |
| LINC01422 | 0.557774 | 1.495281 | 1.422665 | 0.001781 | 0.048778 |
| AC005828.1 | 0.280392 | 0.787413 | 1.489678 | 0.00155 | 0.046732 |
| AL096869.2 | 0.392471 | 0.885013 | 1.173112 | 0.000264 | 0.033712 |
| HMGA2-AS1 | 0.20225 | 2.093771 | 3.371894 | 0.000181 | 0.02988 |
| LINC02848 | 0.292618 | 0.768401 | 1.39284 | 0.001126 | 0.046041 |
| LINC00958 | 0.100638 | 1.787119 | 4.150388 | 0.000357 | 0.035443 |
| AC138207.5 | 7.497986 | 3.478576 | -1.10801 | 0.001896 | 0.048778 |
| AC074254.1 | 0.201062 | 1.425447 | 2.825699 | 0.000563 | 0.039751 |
| AL645608.2 | 1.613259 | 5.947619 | 1.882334 | 3.56E-05 | 0.017652 |
| AC004381.1 | 2.007267 | 4.927638 | 1.295663 | 0.001896 | 0.048778 |
| AL159972.1 | 0.208817 | 0.669334 | 1.680484 | 0.000483 | 0.039751 |
| LINC01238 | 1.623578 | 3.423395 | 1.076252 | 0.001383 | 0.046041 |
| AC092800.1 | 0.23237 | 0.798884 | 1.781564 | 0.000168 | 0.028939 |
| AL606489.1 | 0.908332 | 2.285165 | 1.331006 | 0.000818 | 0.043915 |
| Z97652.1 | 1.076283 | 2.209251 | 1.037499 | 0.001297 | 0.046041 |
| AC093110.1 | 1.880614 | 3.952879 | 1.0717 | 0.000227 | 0.031106 |
| AL360169.1 | 0.619923 | 1.311336 | 1.080877 | 0.001625 | 0.046732 |
| NCOA7-AS1 | 0.468148 | 0.959746 | 1.035686 | 0.001189 | 0.046041 |
| AC006213.4 | 1.481257 | 3.012692 | 1.024231 | 0.001474 | 0.046041 |
| AC027020.2 | 1.859601 | 3.731718 | 1.004847 | 0.000329 | 0.035287 |
| AC012464.1 | 0.44997 | 1.003818 | 1.157598 | 0.000168 | 0.028939 |
| TTC3-AS1 | 0.637005 | 1.314961 | 1.045644 | 9.14E-06 | 0.014592 |
| AC027020.1 | 1.187878 | 2.63372 | 1.148716 | 0.00038 | 0.035929 |
| AL137025.1 | 0.155585 | 0.772022 | 2.310937 | 0.000581 | 0.039751 |
| AC107308.1 | 0.010614 | 0.703076 | 6.049641 | 0.000207 | 0.02988 |
| AC007285.2 | 0.262678 | 0.752475 | 1.518348 | 0.000124 | 0.027342 |
| ZNF528-AS1 | 1.445946 | 3.079226 | 1.090553 | 0.001571 | 0.046732 |
| AC073534.2 | 0.891587 | 2.11403 | 1.245549 | 0.000853 | 0.043915 |
| SLC12A9-AS1 | 0.650341 | 1.306688 | 1.006647 | 0.001139 | 0.046041 |
| AL096869.1 | 0.594266 | 1.236744 | 1.057366 | 0.000123 | 0.027342 |
| AF127577.2 | 1.719116 | 4.336772 | 1.334955 | 0.000714 | 0.043322 |
| AC015967.1 | 0.491848 | 1.028897 | 1.064813 | 0.000754 | 0.043322 |
| AC138207.1 | 2.012923 | 0.778815 | -1.36994 | 0.000427 | 0.037638 |
| AC011472.1 | 0.358033 | 0.934964 | 1.38482 | 0.000732 | 0.043322 |
| AC107079.1 | 0.212998 | 0.660393 | 1.632486 | 4.81E-05 | 0.021198 |
| AC004160.1 | 0.14751 | 0.595604 | 2.013544 | 0.000903 | 0.04468 |
| OVCH1-AS1 | 0.348059 | 1.071002 | 1.621559 | 0.001369 | 0.046041 |
| LINC02593 | 1.077116 | 3.539501 | 1.716372 | 2.51E-05 | 0.015526 |
| AC079766.1 | 0.451999 | 0.970323 | 1.102144 | 0.001189 | 0.046041 |
| AL512413.1 | 0.747864 | 1.972027 | 1.398832 | 0.001461 | 0.046041 |
| AL031005.1 | 0.402046 | 0.977576 | 1.28185 | 0.00142 | 0.046041 |
| AL645608.4 | 0.516769 | 1.842018 | 1.833694 | 8.00E-05 | 0.024412 |

**Abbreviation:**false discovery rate(fdr)，fold change(FC)
