# Supplementary material for: DesA Prognostic Risk Model of LncRNAs in Patients With Acute Myeloid Leukaemia Based on TCGA Data
Source: Front Bioeng Biotechnol. 2022 Feb 21;10:818905. doi: 10.3389/fbioe.2022.818905 (PMC8899517; doi:10.3389/fbioe.2022.818905)
Supplement: Supplementary file 2 [file Table2.docx]

**Supplementary table-2：**the result of multivariate Cox regression analysis

| id | coef | HR | HR.95 Low | HR.95 High | p-value |
| --- | --- | --- | --- | --- | --- |
| LINC01436 | 0.0704 | 1.0729 | 1.007080981 | 1.143104363 | 0.0294 |
| AC073534.2 | -3.033 | 0.7384 | 0.593246829 | 0.919008618 | 0.0066 |
| LINC02593 | -0.1392 | 0.87002 | 0.787491537 | 0.961193092 | 0.0062 |

**Abbreviation:** Identity document (id), Hazard Ratio (HR), coefficient (coef)
