# Supplementary material for: DesA Prognostic Risk Model of LncRNAs in Patients With Acute Myeloid Leukaemia Based on TCGA Data
Source: Front Bioeng Biotechnol. 2022 Feb 21;10:818905. doi: 10.3389/fbioe.2022.818905 (PMC8899517; doi:10.3389/fbioe.2022.818905)
Supplement: Supplementary file 3 [file Table3.docx]

**Supplementary table-3：** Clinical characteristics of patients from TCGA when constructing the model

| Covariates | Type | Total | Test | Train | Pvalue |
| --- | --- | --- | --- | --- | --- |
| age | < 60 | 75 | 39 | 36 | 0.7197 |
|  | ≥ 60 | 53 | 25 | 28 |  |
| gender | Female | 60 | 27 | 33 | 0.3758 |
|  | Male | 68 | 37 | 31 |  |
| FAB subtype | M3 | 13 | 9 | 4 | 0.2418 |
|  | non-M3 | 115 | 55 | 60 |  |
| Mutation frequency | High | 49 | 17 | 32 | 0.1140 |
|  | Low | 39 | 23 | 16 |  |
|  | Other | 40 | 24 | 16 |  |
| life status | Alive | 51 | 29 | 22 | 0.2063 |
|  | Dead | 77 | 35 | 42 |  |

**Abbreviation:** FAB: French, American and British; M3: acute promyelocytic leukemia

**Annotation:** High Mutation frequency: Single sample mutation counts ≥ median mutation counts; Low Mutation frequency: single sample mutation counts < median mutation counts. Other: No mutation data. (TCGA- Acute Myelocytic Leukemia data contains mutation data of 134 patients and complete clinical information of 128 patients, taking the intersection of them, there are 88 patients with complete mutation data and clinical information)
